# Alcohol Misuse and Sexually Transmitted Infections: Using the CAGE Questionnaire as a Screening Tool

**DOI:** 10.31486/toj.23.0141

**Published:** 2024

**Authors:** Leah Feulner, Kelly Kossen, Jill Lally, Montana Ellis, Jeff Burton, David Galarneau

**Affiliations:** ^1^ The University of Queensland Medical School, Ochsner Clinical School, New Orleans, LA; ^2^ Center for Outcomes and Health Services Research, Ochsner Clinic Foundation, New Orleans, LA; ^3^ Department of Psychiatry, Ochsner Clinic Foundation, New Orleans, LA

**Keywords:** *Alcohol drinking*, *chlamydia*, *gonorrhea*, *hepatitis*, *HIV*, *sexually transmitted diseases*, *syphilis*, *trichomonas infections*

## Abstract

**Background:** While the connection between alcohol and risky behavior is well known, a clear correlation between alcohol misuse and contracting sexually transmitted infections (STIs) has not been determined. The 4-question CAGE questionnaire—the acronym stands for attitudes and activities related to alcohol use—is often administered at primary care annual visits to screen patients for alcohol abuse. This study assessed the relationship between CAGE scores and STI results to determine if the CAGE questionnaire could help determine the need for STI screening at annual visits.

**Methods:** All patients who received a CAGE screening from 2015 to 2022 at a Gulf South health system were included in the analysis. The primary outcome of the study was the relationship between a positive CAGE score (a score ≥2) and a positive STI result. STIs included in the primary analysis were human immunodeficiency virus (HIV), hepatitis B, syphilis, chlamydia, gonorrhea, and trichomoniasis. The correlation between a positive CAGE score and hepatitis C was examined as a secondary outcome.

**Results:** A total of 40,022 patients received a CAGE screening during the study period, and 757 (1.9%) scored ≥2 on the CAGE questionnaire. Significant associations were found between a positive CAGE score and hepatitis B (odds ratio [OR]=2.69, 95% CI 1.91, 3.80; *P*<0.001), gonorrhea (OR=5.43, 95% CI 1.80, 16.39; *P*=0.003), and hepatitis C (OR=2.10, 95% CI 1.57, 2.80; *P*<0.001). No associations were found between a positive CAGE score and HIV, chlamydia, or trichomoniasis. No patients with a CAGE score ≥2 had a syphilis diagnosis; therefore, no syphilis analysis was possible.

**Conclusion:** Based on the results of this study, patients with a CAGE score ≥2 may benefit from screening for hepatitis B, hepatitis C, and gonorrhea at their primary care annual visit. Early STI detection could lead to prompt treatment and prevent further transmission and complications.

## INTRODUCTION

### Preventive Screenings

The primary function of preventive health screenings during primary care visits is to identify diseases in their initial stages, and thus allow health care professionals to provide early treatment and mitigate consequences.^1^ Annual primary care visits are recommended for all patients so the provider can track the patient's health, including cardiac and diabetic risks, and provide treatment and/or referrals. The United States Preventive Services Task Force (USPSTF), a panel of experts in disease prevention and primary care, recommends that preventive services, such as counseling and screenings, should generally be provided in the primary care setting.^2^ Because of the recognized importance of preventive care, the Affordable Care Act mandated that all Marketplace health plans, in addition to many other insurance plans, provide essential services such as blood pressure screening, diet counseling, immunizations, and cancer screening without charging a copayment or coinsurance.^3^ All the grade A and B recommendations from the USPSTF are available at no cost; these are the services the USPSTF designated as highly important for eligible patients.^4^

Because of the health risks of heavy alcohol use, the USPSTF recommends that all adults 18 years and older be screened via questionnaire for alcohol use at primary care visits.^5^ Although patients are often asked about their drug and alcohol use, screening via a questionnaire is not routinely done. Chatterton et al determined that alcohol screening via a questionnaire was only performed at 2.6% of primary care visits between 2014 and 2016.^6^

### CAGE Questionnaire

The CAGE questionnaire was first introduced in 1970 by its creators, Drs Ewing and Rouse, and is still used to screen for alcohol use.^7^ The CAGE questionnaire gives providers a way to assess a patient's drinking habits in a relatively nonconfrontational manner. The questionnaire consists of 4 questions and is scored by assigning 1 point for every yes response. Higher scores indicate a greater risk for alcohol misuse.^8^ CAGE is an acronym that stands for the attitudes and activities assessed by 4 questions: cutting down, annoyed by criticism, guilty feeling, and [using] eye-openers. The actual questions are shown in Table 1. Bernadt et al found that the CAGE questionnaire was effective in identifying 9 of 10 individuals with alcoholism.^9^ Fiellin et al found the CAGE questionnaire to be more effective in detecting alcohol abuse and dependence than other alcohol screening questionnaires but found the Alcohol Use Disorders Identification Test (AUDIT) to be superior at identifying at-risk or harmful drinking.^10^

**Table 1.** CAGE Questionnaire^7,8^

		Responses and Scoring	
Question Abbreviation	Question	Yes	No
C	Have you ever felt you should cut down on your drinking?	1	0
A	Have people annoyed you by criticizing your drinking?	1	0
G	Have you ever felt bad or guilty about your drinking?	1	0
E	Have you ever had a drink first thing in the morning to steady your nerves or to get rid of a hangover?	1	0

### Alcohol Use and Sexually Transmitted Infections

The negative impacts of alcohol abuse have long been identified and include mental disorders such as anxiety and depression; chronic diseases such as high blood pressure, heart disease, liver disease, and stroke; and risky sexual behavior.^11,12^

In 2021, 29.5 million people in the United States older than 12 years (10.6%) struggled with alcohol use disorder.^13^ While the concept that alcohol use leads to risky behavior is not novel, quality evidence supporting the claim that heavy alcohol use is associated with increased rates of sexually transmitted infections (STIs) is lacking. A clear correlation between alcohol consumption and patterns of sexual behavior has been difficult to establish because of methodology limitations and a lack of meaningful cross-sectional studies. However, a 2016 meta-analysis and a 2002 systematic review found positive correlations between alcohol use and willingness to engage in unprotected sex with potential partners.^14,15^ Other studies have shown that unprotected sex increases the likelihood of acquiring a sexually transmitted infection.^16,17^ Alcohol use has also been shown to be associated with a history of multiple sexual partners, another factor that increases the likelihood of acquiring a sexually transmitted infection.^15,18-20^

Screening for STIs is important as many STIs can be asymptomatic, and the World Health Organization estimates that 1 million STIs are acquired every day.^21^ The 4 curable STIs—syphilis, gonorrhea, chlamydia, and trichomoniasis—can generally be easily treated with 1-time doses of antibiotics.^21^ However, detection can be challenging. Approximately 48% of women will have no symptoms with a gonorrhea infection, and 64% to 68% of men remain asymptomatic.^22-24^ Gonorrhea and chlamydia can have serious consequences if left untreated, such as pelvic inflammatory disease, a condition that can affect fertility.^25^ Syphilis can progress to neurologic problems and death if left untreated.^26^

Incurable STIs include hepatitis B, herpes simplex virus, human immunodeficiency virus (HIV), and human papillomavirus. Hepatitis B is not endemic to the United States, but infections in the United States have increased because of immigration from endemic regions. Although a hepatitis B vaccine is available, 5% of people will have an inadequate immune response, and 10% will not respond even on revaccination attempts.^27^ Early treatment of hepatitis B has been shown to reduce premature deaths and delay disease progression.^28^ Risky sexual behavior could lead to increased rates of hepatitis B in part because of the increased likelihood of engaging in anal intercourse.^29-31^

HIV is characteristically asymptomatic and often presents with vague symptoms such as a flu-like prodrome. In a 2016 study, as few as 29% of people infected with HIV reported symptoms.^32^

Heavy alcohol use may increase the risk of contracting an STI because of sexual risk-taking behavior.^14,33^ However, no studies have used standard alcohol use assessments such as the CAGE questionnaire or AUDIT in conjunction with laboratory-confirmed STI testing to test for a relationship between alcohol use and STIs. A 2011 study in Kenya correlated AUDIT scores and sexual risk-taking behavior.^34^ A 2005 systematic review, which analyzed 42 studies, found a significant positive association in 8 of the studies between the level of alcohol consumption and STIs that did not vary by sex or alcohol consumption patterns.^35^ Studies have found a positive correlation between higher CAGE scores and HIV risk.^36-38^ However, all of these studies were conducted outside of the United States in areas where HIV has a higher prevalence. Therefore, studying the US population may be beneficial to determine if higher CAGE scores are associated with higher rates of specific STIs or other comorbid conditions.

This study assessed the relationship between CAGE scores and positive STI results to determine whether the CAGE questionnaire can appropriately identify patients who need STI screening at their annual wellness visit. Given the association of heavy alcohol use with sexually risky behaviors and sexually risky behaviors with STIs, we hypothesized that increased alcohol use as demonstrated by CAGE scores would be associated with an increased rate of STIs.

## METHODS

Institutional review board approval was obtained prior to acquiring and investigating patient data.

### Study Design

We conducted an initial literature search to identify any preexisting correlations between STIs and positive CAGE scores to determine if a similar study had been done. Patient data were obtained from the Ochsner Health Epic (Epic Systems Corporation) electronic health record (EHR). Ochsner Health is a tertiary health system that serves the states of Louisiana and Mississippi and the Gulf South. Ochsner Health has 46 hospitals and more than 370 health centers and served 1.5 million patients in 2023.^39^ The Ochsner Health EHR contains information on more than 6.2 million patients, but only approximately 3 million patient files met the following criteria for our Patient-Centered Outcomes Research Institute (PCORI) Common Data Mart (CDM): patients must be in the official Ochsner Health service area (which includes New Orleans, Lafayette, and Baton Rouge, Louisiana, and southern Mississippi) and have at least 1 in-person encounter in the last 5 years.^40-42^ The PCORI CDM was queried using Oracle SQL Developer, version 19.2.0.206 (Oracle).

Records for all patients who received a CAGE screening between 2015 and 2022 were obtained, and we examined the patient records for positive STI results. The presence of an STI was confirmed by any corresponding diagnosis or positive laboratory test (Table 2) documented in the EHR. STIs included in our analysis were HIV, hepatitis B, syphilis, chlamydia, gonorrhea, and trichomoniasis. Herpes simplex virus 1 and 2 were excluded because the Centers for Disease Control and Prevention (CDC) guidelines do not recommend testing patients without symptoms.^43^

**Table 2.** Sexually Transmitted Infection Diagnostic Tests

Sexually Transmitted Infection	Laboratory Test
HIV	HIV antigen/antibody test
Hepatitis B	Hepatitis B surface antigen test
Syphilis	Rapid plasma reagin test, Venereal Disease Research Laboratory test
Chlamydia	Chlamydia nucleic acid amplification test
Gonorrhea	Gonorrhea nucleic acid amplification test
Trichomoniasis	Trichomoniasis nucleic acid amplification test
Hepatitis C	Hepatitis C antibody test

HIV, Human immunodeficiency virus.

The primary outcome of the study was the correlation between a positive CAGE score and a positive STI result.

We examined the correlation between a positive CAGE score and hepatitis C as a secondary outcome. Hepatitis C was included as a secondary outcome because although not common, hepatitis C can be transmitted through sexual contact, and studies have identified a correlation between alcohol misuse and hepatitis C.^44,45^ CDC guidelines recommend that all patients older than 18 years have a 1-time hepatitis C screening.^46^

### Statistical Analysis

Continuous measures are presented as means and standard deviations. Categorical measures are presented as frequencies and percentages.

For patients with multiple completed CAGE questionnaires, the highest documented score was used for analysis. A score ≥2 was defined as a clinically significant positive result. We used this score cutoff because Malet et al reported a sensitivity of 77% and specificity of 94% for alcohol use disorder in patients scoring ≥2.^47^

Logistic regression models were used to assess associations between CAGE questionnaire results and infections. Each model contains 1 explanatory variable and a univariate binary response. The explanatory variables investigated are total CAGE scores and binary indicators of total CAGE scores ≥2. The univariate binary response in each regression model represents the presence/absence of either an individual infection or a predefined grouping of infections. The predefined groupings of infections were *any STI (*HIV, hepatitis B, syphilis, chlamydia, gonorrhea, and trichomoniasis); *STI (blood screen)* (HIV, hepatitis B, syphilis); and *STI (non-blood screen)* (chlamydia, gonorrhea, trichomoniasis). Because of the rarity of documented infections in the sample, the Firth bias-reducing penalized likelihood method was used for estimating model parameters. Estimated odds ratios (ORs) were calculated for individual infections and for predefined groupings of infections. For each test, the null hypothesis is H_0_: OR=1.0, and the alternative hypothesis is H_1_: OR≠1.0. All tests are evaluated at significance level of 0.05. Estimated ORs are presented with 95% CIs, and statistical significance is determined by a CI that does not contain the null value 1.0.

Where applicable, *P* values and CIs were adjusted for multiple comparisons via simulation. Analyses were conducted using SAS (SAS Institute Inc) version 9.4 for Windows (Microsoft Corporation).

## RESULTS

### Demographics

Our sample consisted of 40,022 Ochsner Health patients from Louisiana and Mississippi. Demographics, clinical characteristics, and STI diagnoses for the patient sample are summarized in Table 3. Patient age ranged from 21 to 105 years, with a mean age of 71.9 years. The majority of the patients were female (57.1%), and 64.4% of the population was White.

**Table 3.** Patient Characteristics, n=40,022

Variable	Value
**Demographics**	
Age, years, mean (SD)	71.9 (9.50)
Age, years, median [IQR]	72 [67-78]
Age, years, range	21-105
Female	22,839 (57.1)
Race/Ethnicity	
Black, non-Hispanic	12,687 (31.7)
White, non-Hispanic	25,762 (64.4)
Hispanic	897 (2.2)
Other/unknown	676 (1.7)
Marital status	
Married/significant other	21,467 (53.6)
Divorced/separated	4,419 (11.0)
Widowed	8,698 (21.7)
Single	5,339 (13.3)
Other/unknown	99 (0.3)
Insurance	
Commercial	10,233 (25.6)
Medicare	28,801 (72.0)
Medicaid	280 (0.7)
Other	113 (0.3)
Uninsured/self-pay	154 (0.4)
Unknown	441 (1.1)
**Clinical characteristics**	
Heart rate, beats per minute, mean (SD)	73.7 (10.7)
Body mass index, kg/m^3^, mean (SD)	29.7 (6.5)
Weight, lbs, mean (SD)	185.6 (46.0)
**Sexually transmitted infection diagnosis**	
Human immunodeficiency virus	125 (0.3)
Hepatitis B	738 (1.8)
Syphilis	199 (0.5)
Chlamydia	50 (0.1)
Gonorrhea	36 (0.1)
Trichomoniasis	473 (1.2)
Hepatitis C^a^	1,369 (3.4)

^a^Secondary outcome.

Notes: Sexually transmitted infection diagnoses were derived using a combination of documented *International Classification of Diseases-10* codes and results from clinical laboratory testing. Data are presented as n (%) unless otherwise indicated.

### Primary Outcome: Correlation Between Positive CAGE Score and Positive STI Result

Of the 40,022 patients in the sample, 757 (1.9%) scored ≥2 on a CAGE questionnaire (Table 4). Table 5 shows the number of patients with each STI, stratified by positive and negative CAGE scores.

**Table 4.** CAGE Questionnaire Scores, n=40,022

Total CAGE Score	n (%)
0	38,170 (95.4)
1	1,095 (2.7)
2	437 (1.1)
3	204 (0.5)
4	116 (0.3)
Patients with score ≥2	757 (1.9)

**Table 5.** Sexually Transmitted Infections by Positive and Negative CAGE Scores, n=40,022

	Total CAGE Score ≥2	
Sexually Transmitted Infection	Yes, n=757	No, n=39,265
HIV	1 (0.1)	124 (0.3)
Hepatitis B	35 (4.6)	703 (1.8)
Syphilis	0	199 (0.5)
Chlamydia	1 (0.1)	49 (0.1)
Gonorrhea	3 (0.4)	33 (0.1)
Trichomoniasis	9 (1.2)	464 (1.2)
Hepatitis C^a^	51 (6.7)	1,318 (3.4)
STI (blood screen)^b^	35 (4.6)	951 (2.4)
STI (non-blood screen)^c^	13 (1.7)	523 (1.3)
Any STI^d^	48 (6.3)	1,437 (3.7)

^a^Secondary outcome.

^b^STI (blood screen) = HIV, hepatitis B, syphilis.

^c^STI (non-blood screen) = chlamydia, gonorrhea, trichomoniasis.

^d^Any STI = HIV, hepatitis B, syphilis, chlamydia, gonorrhea, trichomoniasis.

Notes: A positive—clinically significant—CAGE score was defined as ≥2, and a score <2 was defined as negative. Each individual infection for patients with multiple infections is counted, but each patient is only counted once for STI (blood screen), STI (non-blood screen), and Any STI.

HIV, human immunodeficiency virus; STI, sexually transmitted infection.

A positive CAGE screen (≥2) when compared to a CAGE score of 0 was positively associated with hepatitis B and gonorrhea (Table 6). Compared to patients with a CAGE score of 0, patients with positive CAGE screens had 169% (OR=2.69, 95% CI 1.91, 3.80; *P* <0.001) and 443% (OR=5.43, 95% CI 1.80, 16.39; *P*=0.003) higher odds of a positive hepatitis B surface antigen and a positive gonorrhea nucleic acid amplification test, respectively (Table 6). Patients with a CAGE score ≥2 also had 80% (OR=1.80, 95% CI 1.34, 2.42; *P* <0.001) higher odds of a positive result for any STI (HIV, hepatitis B, syphilis, chlamydia, gonorrhea, or trichomoniasis). No statistically significant associations were found between a positive CAGE score and a positive test for HIV, chlamydia, or trichomoniasis. Syphilis could not be analyzed because of a zero occurrence rate among patients with a CAGE score ≥2 (Table 5).

**Table 6.** Odds Ratios for Sexually Transmitted Infections in Patients With a Total CAGE Score ≥2

Sexually Transmitted Infection	Odds Ratio (95% CI)	*P* Value
HIV	0.62 (0.12, 3.13)	0.566
Hepatitis B	**2.69 (1.91, 3.80)**	**<0.001**
Syphilis	NE	–
Chlamydia	1.57 (0.31, 7.99)	0.586
Gonorrhea	**5.43 (1.80, 16.39)**	**0.003**
Trichomoniasis	1.06 (0.56, 2.02)	0.859
Hepatitis C^a^	**2.10 (1.57, 2.80)**	**<0.001**
Any STI^b^	**1.80 (1.34, 2.42)**	**<0.001**

^a^Secondary outcome.

^b^Any STI = HIV, hepatitis B, syphilis, chlamydia, gonorrhea, trichomoniasis.

Note: Results are presented from comparisons of CAGE score ≥2 vs CAGE score = 0. Values in bold are statistically significant.

HIV, human immunodeficiency virus; NE, not estimable (because of zero occurrences in the CAGE score ≥2 group); STI, sexually transmitted infection.

### Secondary Outcome: Correlation Between Positive CAGE Score and Positive Hepatitis C Result

Of the 757 patients scoring ≥2 on a CAGE questionnaire, 51 tested positive for hepatitis C (Table 5), and the association between a positive CAGE score and hepatitis C was statistically significant. Compared to patients with a CAGE score of 0, patients with positive CAGE screens had 110% higher odds of a positive hepatitis C antibody test (OR=2.10, 95% CI 1.57, 2.80; *P*<0.001) (Table 6).

## DISCUSSION

### Hepatitis B

Patients who scored ≥2 on the CAGE questionnaire had almost 3 times the odds of having hepatitis B compared to patients who scored 0. Although treatment for acute hepatitis B is supportive, after detection, careful monitoring of the viral load at primary care visits can determine if the virus has been cleared and if treatment for chronic hepatitis B should be started. Earlier care has been associated with decreased viral load, which lowers the risks of decompensated cirrhosis, hepatocellular carcinoma, and transplant.^28^ Alcohol-induced hepatitis B can cause an increase in hepatitis B replication, oxidative stress, and suppression of the immune response, ultimately leading to liver fibrosis and development of hepatocellular carcinoma.^48,49^ Alcohol abusers have more rapid disease progression and an increased rate of chronic hepatitis B compared to nonheavy alcohol users.^48^ Although the vaccine for hepatitis B has significantly decreased disease incidence, approximately 5% of the population remains classified as nonresponders, and response to the vaccine is variable among people with chronic disease.^27^ If a patient has a positive CAGE result, primary care providers should emphasize the high risk of concurrent alcohol use and hepatitis B infection when discussing alcohol cessation and hepatitis B testing.

### Gonorrhea

Gonorrhea had the strongest association with a positive CAGE score. Patients who scored ≥2 on the CAGE questionnaire had 5 times higher odds of testing positive for gonorrhea compared to patients who scored 0. Two studies examining the association between alcohol consumption and gonorrhea, syphilis, and chlamydia found significant associations only between gonorrhea and alcohol use.^29,50^ Hutton et al examined men and women in an STI clinic and found that gonorrhea was 5 times higher in binge-drinking women compared to women abstainers.^29^ An Australian study found that people who abuse alcohol had a 1.6 times higher risk of contracting gonorrhea compared to people who did not abuse alcohol.^50^ Given the consequences of untreated gonorrhea and the relative ease of treatment,^51^ the CAGE questionnaire could be used as a tool for identifying people at risk who may benefit from further screening to receive earlier treatment.

### Hepatitis C

Hepatitis C was the most prevalent infection in our population, and the odds of having hepatitis C were 2 times higher in patients who scored ≥2 on the CAGE questionnaire. The relationship between hepatitis C and alcohol has been studied, but no clear consensus has been reached as to why heavy alcohol consumption is associated with higher rates of hepatitis C. Pessione et al reported a significant correlation between grams of ethanol ingested and hepatitis C RNA levels.^52^ Rosman et al suggested that alcohol-induced liver inflammation possibly predisposes alcohol users to hepatitis C infections even when other predisposing factors such as blood transfusion or intravenous drug use are absent.^53^ Unlike hepatitis B, hepatitis C does not have a vaccine but has a curable treatment that has proven effective in patients with alcohol use disorder.^54^ Faster treatment initiation can limit the progression of disease, improve treatment outcomes, and prevent transmission.^55^ Although the CDC only recommends a 1-time screen for hepatitis C in adults 18 years and older, periodic screening for patients with risk factors is still recommended.^56^ A positive CAGE score could prompt primary care providers to increase the frequency of screening for hepatitis C.

### Limitations

Our study has several limitations. In the general population, approximately half the HIV, syphilis, chlamydia, gonorrhea, and trichomoniasis cases occur in patients 15 to 24 years old.^57,58^ However, our study population had low pretest probability of STIs because of an average age of 71.9 years. Only approximately 4% of the sample population had diagnosed STIs. The low prevalence of STIs likely contributed to our large CIs and possibly contributed to our lack of significant findings for HIV, syphilis, chlamydia, and trichomoniasis. Additionally, only 1.9% of our population had a CAGE score ≥2, limiting the size of our testable population. Another limitation is that no data were available on the percentage of Ochsner Health patients who were asked to answer the CAGE questionnaire and what types of providers used the CAGE questionnaire with their patients. Therefore, provider practices and selection may have introduced study bias.

### Future Research

Future research could examine the use of the CAGE questionnaire in a population with higher pretest probability such as at adolescent health clinics and reproductive health clinics. Future research could also evaluate the correlation between the alcohol screening tool AUDIT and STIs because AUDIT has been shown to be a better predictor of active heavy drinking and active alcohol abuse.^10^ A prospective study could screen all patients with clinically significant CAGE scores for STIs to determine further significant correlation. Also, a study requiring providers to administer the CAGE questionnaire to all patients would reduce the potential selection bias.

## CONCLUSION

The CAGE questionnaire may be a useful tool to help identify patients who should be screened for STIs. The correlation between a positive CAGE score and STIs may also prompt primary care providers to increase their use of the CAGE questionnaire. Patients with a CAGE score ≥2 may benefit from screening for hepatitis B, hepatitis C, and gonorrhea at their primary care visits to receive earlier intervention.

## Notes

*This article meets the Accreditation Council for Graduate Medical Education and the American Board of Medical Specialties Maintenance of Certification competencies for Patient Care and Medical Knowledge.*
